# Evaluation of Two PCR Tests for *Coxiella burnetii* Detection in Dairy Cattle Farms Using Latent Class Analysis

**DOI:** 10.1371/journal.pone.0144608

**Published:** 2015-12-16

**Authors:** Simon Nusinovici, Aurélien Madouasse, Thierry Hoch, Raphaël Guatteo, François Beaudeau

**Affiliations:** 1 INRA, UMR1300 Biology, Epidemiology and Risk Analysis in animal health, CS 40706, F-44307, Nantes, France; 2 LUNAM Université, Oniris, UMR BioEpAR, CS 40706, F-44307, Nantes, France; Auburn University, UNITED STATES

## Abstract

Different tests performed on bulk tank milk samples (BTM) are available to determine the *C*. *burnetii* status of herds. However, these tests, which are based on the detection of either antibodies directed against *C*. *burnetii* (ELISA) or bacterial DNA (PCR), have limitations. A currently disease-free herd infected in the past may continue to test positive with ELISA due to the persistence of antibodies in animals that were infected and that subsequently cleared the infection. Infectious herds can also be misclassified using PCR because of the absence of bacteria in the BTM when the test is performed. Recently, PCR has been used for bacterial DNA detection in the farm environment, which constitutes the main reservoir of *C*. *burnetii*. The objectives of this study were to assess and compare the sensitivities and specificities of one commonly used PCR test in BTM (PCR BTM) and of a PCR applied to environmental samples (PCR DUST) in dairy cattle farms. BTM and dust samples were collected (using environmental swabs) in 95 herds. The evaluation of the performance of the 2 tests was conducted using latent class models accounting for within herd disease dynamics. Parameter estimation was carried out using MCMC, within a Bayesian framework. Two types of priors were used for the specificity of PCR DUST. A model with a uniform prior on 0–1 fitted the data better than a model with a uniform prior on 0.95–1. With the best model PCR DUST had a lower sensitivity than PCR BTM (0.75 versus 0.83) and a specificity of 0.72. The moderately low value for the specificity of PCR DUST suggests that the presence of bacteria on farm is not always associated with persistent infections and shedding of bacteria in milk.

## Introduction

*Coxiella burnetii* (*C*. *burnetii*) is the infectious agent responsible for Q fever, a zoonosis with worldwide distribution (with the exception of New Zealand). Infection in humans is usually asymptomatic but can induce acute or chronic disease [1]. In livestock *C*. *burnetii* infection can lead to abortion, stillbirth and fertility disorders [2]. *C*. *burnetii* is shed through birth products, feces, urine, milk and vaginal mucus [3–5]. *C*. *burnetii* infection in humans and livestock occurs mainly after inhalation of contaminated aerosols, with shedding ruminants considered as the main reservoir for transmission to humans [6]. The detection of *‘contaminated farms’*, where *C*. *burnetii* is present, could therefore help to prevent infection in humans.

Different tests are available to determine the *C*. *burnetii* status of herds. These tests are based on either the detection of antibodies directed against *C*. *burnetii* (ELISA) or of bacterial DNA (PCR). As bulk tank milk (BTM) provides an aggregated measure at the herd level and is easy to collect, it is frequently the target sample. ELISA tests applied to BTM are cheap and thus used in epidemiological studies. However, these tests are not well suited for detecting infectious farms because a positive test result can be due to persistent antibodies from past infection. PCR tests performed in BTM allow the detection of *C*. *burnetii* shedder cows. However, falsely negative responses may occur as infectious cows can shed bacteria in milk intermittently [5], or when the shedder cows are dried-off.

*C*. *burnetii* is highly resistant in the environment which therefore constitutes the main source of contamination for both humans and animals. Recently tests based on the detection of bacterial DNA in the environment have been developed. As a result *C*. *burnetii* has been detected in airborne dust samples and surface area swabs in BTM-positive goat farms during an outbreak in the Netherlands [7–9]. Such environmental samples could thus be a complementary option to assess a herd’s *C*. *burnetii* status regarding the presence of the bacteria. To our knowledge, the sensitivity and specificity of tests based on DNA detection have not yet been evaluated in either environmental samples or BTM.

The difficulty associated with such an evaluation is that there is no reference test, i.e. gold standard, against which to compare the outcomes of environmental and BTM PCRs. There exists a rich literature on modelling disease status and test outcomes in the absence of a gold standard [10]. Most of the work in this area builds on an article by Hui and Walter (1980) who derived a method for estimating disease prevalences and test characteristics in the absence of a gold standard when several tests were available [11]. The numbers of populations and tests required for the estimation procedure to be accurate were constrained by the number of parameters to estimate. Further assumptions were that the tests had the same characteristics in all populations and that the test results were conditionally independent. Using Bayesian methods [12], it becomes possible to relax the constraints on the numbers of populations and tests by putting priors on parameters for which some information is already available [12,13]. A further improvement consists in modelling the covariances between test results [12]. Finally, with longitudinal data, the latent statuses at consecutive points in time may be correlated. Incorporating a time correlation in the latent states should therefore lead to a more accurate estimation of model parameters [14–16].

The objectives of this study were to assess and compare the sensitivities and specificities of one commonly used PCR test in BTM and of a PCR test applied to environmental samples in dairy cattle farms using latent class analysis. Both single tests and a combination of tests, used in parallel or in series, were evaluated.

### Ethics Statement

Except the permission of the farmers who kindly accept to participate to this study, no specific permissions were required for the data collection. Moreover, this study did not involve endangered or protected species.

## Materials and Methods

### Sample collection

**Fig 1:**
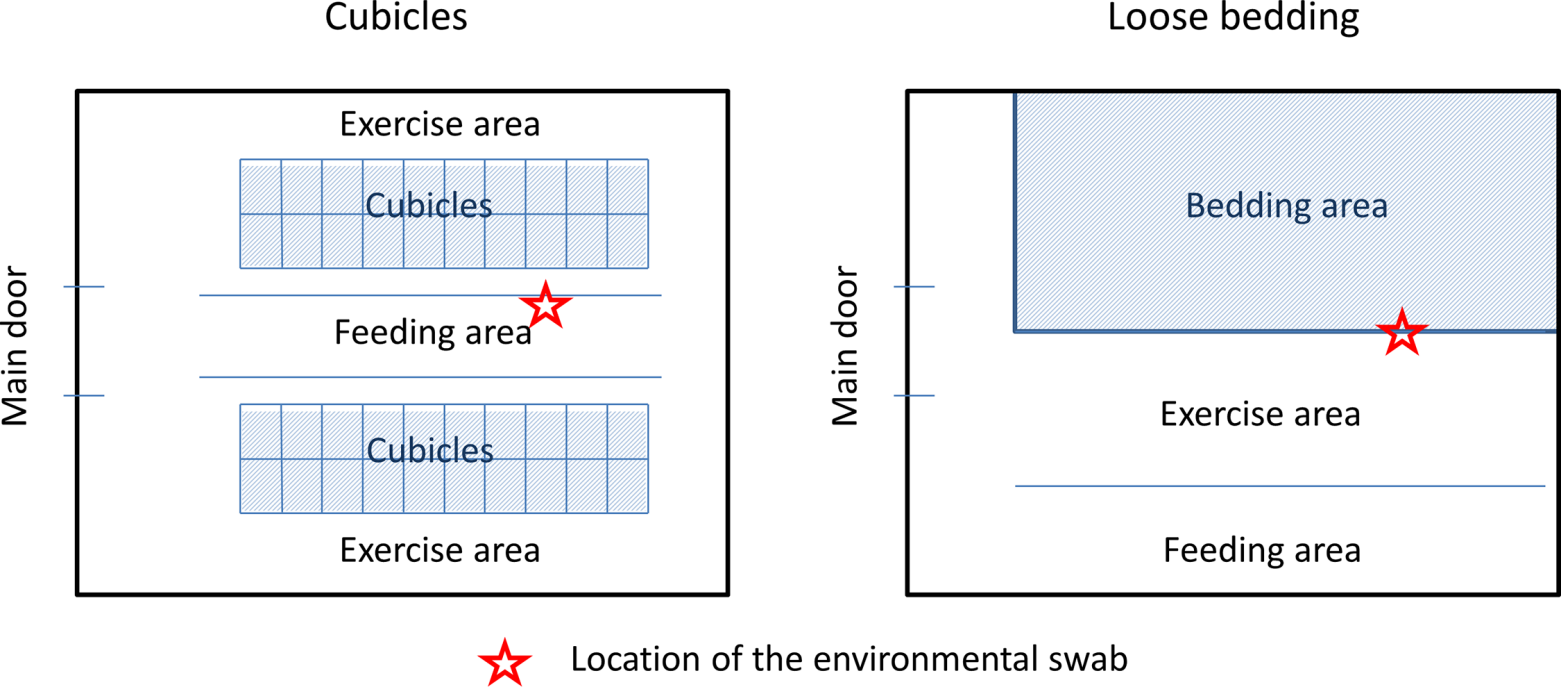
Location of environmental swabs according to the main types of barn.

Data were collected in the Finistère department located in the north western part of France. Ninety-five dairy cattle herds were selected in different parts of the department. These herds were followed for a total period of 1 year. In each herd, BTM samples and indoor dust were collected every 4 months. There were thus 4 sampling times for each herd. Passive accumulation of dust was collected using environmental swabs placed in the barn for a 2-week period. Swab samplings were standardized between herds towards the likelihood of detecting *C*. *burnetii*. Swab locations were chosen to minimize the distance to cows and maximize the distance to the main door as shown in Fig 1. In addition, data collection was calibrated between operators in order to ensure homogeneity of the collection process.

### Diagnostic tests

Two diagnostic tests were performed at the herd level:

- PCR BTM: Each BTM sample was tested for *C*. *burnetii* presence using a commercial kit (TaqVet Coxiella burnetii–Absolute Quantification, LSI, Lissieu, France), targeting IS1111 transposase, following the manufacturer’s instructions. The external positive control used was a solution containing 3.10^7^ number of gene copies per mL. The negative control sample used was a DNase RNase free water. DNA was extracted using the QIAmp® DNA mini kit (Qiagen S.A., France) following the manufacturer’s instructions. Extraction was performed directly from 200μL of BTM. All PCR assays were performed using ABI 7500 Real Time PCR System (Applied Biosystems, France). For positive samples, i.e. those with a typical amplification curve, results were first given in Ct (cycle threshold) values. Only samples presenting a typical curve with a Ct below 45 were considered to be positive.
- PCR DUST: Each dust sample was tested for *C*. *burnetii* presence using a commercial kit (TaqVet Coxiella burnetii Feces Environment, LSI, Lissieu, France) also targeting IS1111 transposase. The environmental swabs were vortexed with 40 mL of phosphate buffered saline solution (PBS). To avoid false negative results due to low concentration of the bacteria in the swabs, 1.5 mL of the PBS solution was then centrifuged to concentrate the bacteria. Finally, extraction was performed on the centrifugation sediment. As the extraction is usually performed from 200μL of solution, raw results were divided by 7.5 to account for the impact of centrifugation (corresponding to the ratio 1.5mL/200μL) to obtain the total number of bacteria per ml of PBS.

In both cases, the results were expressed in number of *C*. *burnetii/* mL.

### Statistical analyses

#### Models considered

The evaluation of the performance of the 2 tests was conducted using latent class models. Parameter estimation was carried out using MCMC, within a Bayesian framework. This type of analysis allows the evaluation of sensitivity and specificity of one or several tests, in the absence of a gold standard. A latent class model refers to situations where the event of interest is not directly observed, i.e. latent. In this study, what was measured in the farms was the shedding of bacteria in the milk of infected cows (based on PCR applied to BTM) and the environmental contamination (using the PCR applied to dust).

Within the current study design, contrary to the approach followed by Toft et al. (2007), it was difficult to split the available herd population into 2 or more groups with different disease prevalences [17]. On the other hand, the 4 sampling times per herd provided some information on the herd status that can be exploited. In order to account for these constraints, the Hui-Walter model was adapted. The model outcome was one of the 4 possible combinations of two test responses in a herd on a given sampling time, which followed a multinomial distribution. This was modelled as follows:

$$\begin{array}{c} O_{ij}\sim Multinomial(1,p_{ij}^{k})\\ p_{ij}^{1}=p(T_{1}^{+},T_{2}^{+}|S_{ij})\\ p_{ij}^{2}=p(T_{1}^{+},T_{2}^{-}|S_{ij})\\ p_{ij}^{3}=p(T_{1}^{-},T_{2}^{+}|S_{ij})\\ p_{ij}^{4}=p(T_{1}^{-},T_{2}^{-}|S_{ij}) \end{array}$$

Where *O*_*ij*_ was the combined response of test 1 (*T*_*1*_, PCR on BTM) and test 2 (*T*_*2*_, PCR on dust), with 4 possible outcomes: ++, +-, -+ and—, on time *i* in herd *j*. These outcomes occurred with probability $p_{ij}^{1}$ to $p_{ij}^{4}$ respectively which depended on the latent status at time *i* in herd *j*, denoted *S*_*ij*_.

The simplest version of the model assumed conditional independence between tests parameters (CID model). If the outcomes of the2 tests were independent the probabilities $p_{ij}^{1}$ to $p_{ij}^{4}$ could be written as:

$$\begin{array}{l} p_{ij}^{1}=Se_{1}Se_{2}S_{ij}+(1-Sp_{1})(1-Sp_{2})(1-S_{ij})\\ p_{ij}^{2}=Se_{1}(1-Se_{2})S_{ij}+(1-Sp_{1})Sp_{2}(1-S_{ij})\\ p_{ij}^{3}=(1-Se_{1})Se_{2}S_{ij}+Sp_{1}(1-Sp_{2})(1-S_{ij})\\ p_{ij}^{4}=(1-Se_{1})(1-Se_{2})S_{ij}+Sp_{1}Sp_{2}(1-S_{ij}) \end{array}$$

where *Se*_*1*_ and *Se*_*2*_ are the sensitivities of test 1 and test 2 respectively and *Sp*_*1*_ and *Sp*_*2*_ are the specificities of test 1 and test 2 respectively.

Because the outcome of one test could be correlated with the outcome of the other test, the covariance between the 2 sensitivities was modelled (COD model). Since one of the specificities was assumed to be equal to 1 (see below), the covariance between them was not modelled. To accommodate for conditional dependence between sensitivities, the equations were modified as follows:

$$\begin{array}{l} p_{ij}^{1}=(Se_{1}Se_{2}+\gamma_{Se})S_{ij}+(1-Sp_{1})(1-Sp_{2})(1-S_{ij})\\ p_{ij}^{2}=(Se_{1}(1-Se_{2})-\gamma_{Se})S_{ij}+(1-Sp_{1})Sp_{2}(1-S_{ij})\\ p_{ij}^{3}=((1-Se_{1})Se_{2}-\gamma_{Se})S_{ij}+(1-Sp_{2})(1-S_{ij})\\ p_{ij}^{4}=((1-Se_{1})(1-Se_{2})+\gamma_{Se})S_{ij}+Sp_{1}Sp_{2}(1-S_{ij}) \end{array}$$

where *γ*_*Se*_ was the covariance between the 2 sensitivities. Constraints were put on the covariance as described in Toft et al. (2007):

$$max[-(1-Se_{1})(1-Se_{2}),-Se_{1}Se_{2}]\leq \gamma_{Se}\leq min[Se_{1}(1-Se_{2}),(1-Se_{1})Se_{2}]$$

It was hypothesized that the status of farm *j* on time *i-1* could provide some information on the status of herd *j* on time *i*. The variable *S*_*ij*_ was assumed to follow a Bernoulli distribution with parameter $\tau_{S_{(i-1)j}}$:

$$S_{ij}\sim Bernoulli(\tau_{S_{(i-1)j}})$$

where *τ*_0_ was the probability of a new infection occurring on a farm between two consecutive tests and *τ*_1_ was the probability of not eliminating the infection (‘absence of cure’) between two consecutive tests. *τ*_0_ and *τ*_1_ were assigned uniform priors on 0–1. For the first of the 4 sampling times, there was no previous test result to inform *S*_*1j*_. It was assumed that at the start of the study, the disease prevalence was in a state of equilibrium in the population, in which case the number of ‘cures’ equals the number of ‘new infections’. If *π* is the disease prevalence at equilibrium, this means that *τ*_0_(1−*π*) = (1−*τ*_1_)*π*. This allows expressing *π* as:

$$\pi =\frac{\tau_{0}}{\tau_{0}+1-\tau_{1}}$$

The estimated prevalence of the latent state at equilibrium was used as a prior for *S*_*1j*_.

Not accounting for a potential conditional dependence could lead to considerable bias in the estimates of the tests’ properties [18]. Therefore the goodness-of-fit of CID and COD models were compared using deviance.

#### Parameter estimation

Parameters were estimated using MCMC in a Bayesian framework. Uninformative priors (uniform on 0–1) were used for the tests’ sensitivities as well as for the *τ*. parameters, but not for specificities. As specificity decreases, the proportion of false positives increases. Based on experts’ opinion, it was considered that when bacterial DNA was amplified in a sample, there really were *C*. *burnetii* in this sample. Thus, a positive PCR BTM was assumed to indicate with certainty that at least one cow was shedding bacteria in her milk. On the other hand, the significance of amplifying *C*. *burnetii* DNA from the environment was evaluated by testing various priors for the specificity of PCR DUST. Two distributions were tested as priors. In a first model (Model 1), a uniform distribution on 0.95–1 was used to model a high specificity for PCR DUST. This means that when there were no bacteria in the farm, the test could yield at most 5% of positives. However, there could also be situations in which bacteria are present on the farm but do not play any epidemiological role, for instance bacteria transported by wind from another farm which do not subsequently infect any cow. Therefore, in a second model (Model 2), a uniform prior on 0–1 was put on PCR DUST. This prior put no constraints on the probability for a PCR DUST positive sample to be associated with animal shedding.

The models were implemented in OpenBUGS [19], dedicated to Bayesian analysis using Markov Chain Monte Carlo (MCMC) methods. For each model, three chains were run for 10,000 iterations. The first 5,000 iterations were discarded (burn-in). The last five thousand iterations were used for the evaluation of posterior distributions. Convergence of the MCMC chains was assessed using the Gelman-Rubin diagnostic [20]. Convergence was assumed for values below 1.1 [21]. Posterior inference was done by calculating means and 95% posterior credibility intervals (PCI) of all the parameters. Raw data and R code used for analyses are available in the supplementary files (S1 Dataset and S1 R Code).

### Test combinations

Combinations of tests used either in parallel or in serial reading were also evaluated for both Model 1 and Model 2. In serial reading, the combined response is positive only if both tests are positive. In parallel reading, the combined response is positive if one or both results are positive. With the model assuming conditional independence (CID), posterior means of sensitivities and specificities were estimated as follows:

$$\begin{array}{l} Se_{parallel}=1-(1-Se_{PCRBTM})(1-Se_{PCRDUST})\\ Sp_{parallel}=Sp_{PCRBTM}\;*\;Sp_{PCRDUST}\\ Se_{serial}=Se_{PCRBTM}\;*\;Se_{PCRDUST}\\ Sp_{serial}=1-(1-Sp_{PCRBTM})(1-Sp_{PCRDUST}) \end{array}$$

## Results

Sixty-nine percent of the test responses were in agreement between PCR tests (Table 1). Eighteen percent were PCR DUST positive while PCR BTM negative. When responses were divergent, around half less bacteria were found in the positive samples, either in BTM or in dust samples, compared to situations where both responses were positive.

**Table 1:** Binary and quantitative test responses for the PCR tests performed in bulk tank milk (PCR BTM) and indoor dust samples (PCR DUST) according to the stratified population. PCR BTM (n = 380) and PCR DUST (n = 380) tests were performed in 95 dairy cattle herds in the Finistère department, France between November 2012 and April 2014.

Test			Test	
PCR BTM	PCR DUST	Total	PCR BTM	PCR DUST
Binary response (+ positive;—negative)			Quantitative response ^a^ (Q1,median,Q3)	
+	+	146	642,4494,16140	2,6,48
+	-	48	240,1704,10020	-
-	+	70	-	1,3,15
-	-	116	-	-
Total		380	514,3604,14280	1,5,35

^a^ calculated among positive test responses

Several models were tested, which differed by the priors put on the specificities and by the inclusion of a covariance term accounting for conditional dependence between the 2 sensitivities. The covariance term was never significant (the credible interval included 0) and the models had a larger deviance than equivalent models which did not include this variable. Therefore, the 2 final models presented in Table 2 assume conditional independence (CID) between test sensitivities. In Model 1, the assumption was made that PCR DUST returned less than 5% of positives in latent status negative farms. In Model 2, the proportion of false positives with PCR DUST could take any value between 0 and 1. Model 2 had a lower deviance, indicating that not constraining the specificity of PCR DUST resulted in a better model fit. In this model, the posterior mean for the specificity of PCR DUST was 0.72 which means that 28% of truly negative herds would test positive with this test. Model 2 also had a higher posterior mean for the sensitivity of PCR BTM (0.83 vs. 0.71). This makes sense as, compared to Model 1, a higher proportion of PCR DUST positives were false positives and were therefore not ‘missed’ by PCR BTM. In Model 2, both the probability of new infection (0.15 vs 0.18) and the probability of not clearing the infection (0.91 vs. 0.93) were smaller than in Model 1. Although the differences appear to be small, they result in a 10% difference in the estimated infection prevalences. The estimated prevalences (τ_0_/(τ_0_+1- τ_1_)) are 0.72 and 0.62 with Model 1 and Model 2 respectively.

**Table 2:** Posterior means and 95% posterior credibility intervals (PCI) of sensitivities (Se) and specificities (Sp) for the PCR tests performed in bulk tank milk (PCR BTM) and indoor dust samples (PCR DUST), for the 2 models with different priors for the specificity of PCR DUST. PCR BTM (n = 380) and PCR DUST (n = 380) tests were performed in 95 dairy cattle herds in the Finistère department, France between November 2012 and April 2014.

	Model 1 Sp PCR DUST~Unif(0.95, 1)		Model 2 Sp PCR DUST~Unif(0, 1)	
Parameter	Mean	95% PCI	Mean	95% PCI
Se PCR BTM	0.71	[0.64;0.77]	0.83	[0.75;0.91]
Se PCR DUST	0.74	[0.68;0.80]	0.75	[0.69;0.80]
Sp PCR DUST	0.96	[0.95;0.97]	0.72	[0.63;0.81]
*τ*_0_ ^a^	0.18	[0.09;0.29]	0.15	[0.08;0.22]
*τ*_1_ ^b^	0.93	[0.88;0.97]	0.91	[0.84;0.96]
Deviance	724		651	

^a^ probability of the bacteria becoming present on a farm between two consecutive tests

^b^ probability of the bacteria disappearing from a farm between two consecutive tests

The parallel reading allowed the posterior mean of sensitivities to reach 0.92 and 0.96 with Model 1 and Model 2 respectively (Table 3). On the other hand, as expected, the serial reading led to the best specificity (equal to 1 with PCR BTM), whatever the model.

**Table 3:** Posterior means and 95% posterior credibility intervals (PCI) of sensitivities (Se) and specificities (Sp) of the tests combined in parallel or serial reading (2 PCR tests performed in bulk tank milk, PCR BTM, and indoor dust samples, PCR DUST) for the 2 models with different priors for the specificity of PCR DUST. PCR BTM (n = 380) and PCR DUST (n = 380) tests were performed in 95 dairy cattle herds in the Finistère department, France between November 2012 and April 2014.

	Parallel reading		Serial reading	
	Mean	95% PCI	Mean	95% PCI
Model 1				
Se	0.92	[0.88;0.96]	0.52	[0.44;0.68]
Sp	0.96	[0.95;0.97]	1	-
Model 2				
Se	0.96	[0.92;0.99]	0.62	[0.52;0.72]
Sp	0.72	[0.63;0.81]	1	-

## Discussion

Models based on latent class analysis were used to estimate the farm-level performances of 2 PCR tests for the detection of *C*. *burnetii* from BTM and dust samples. From the model that fitted the data best (Model 2), a PCR performed on dust samples had a sensitivity of 0.75 and a specificity of 0.72 while a PCR performed on bulk tank milk, which was assumed to have a perfect specificity, had a sensitivity of 0.83. Since the parameterization was forcing all PCR BTM positive samples to be true positives, the latent state can be defined as *Presence of at least one cow excreting C*. *burnetii in her milk within a farm*. With this model, the estimated prevalence of the latent state was of 0.62. Model 1, in which both tests were associated with a close to perfect specificity, did not fit the data as well. Assuming that a positive PCR was associated with the presence of bacteria in the sample (BTM or DUST), this would imply that the presence of bacteria on a farm is not systematically associated with infectiousness of cows. In this case, the latent state can be defined as *Presence of C*. *burnetii within the farm*. This finding contradicts the classical assumption of extreme infectiousness of *C*. *burnetii* [22].

From Table 2 the discrepant responses PCR DUST positive—PCR BTM negative were more frequent than PCR DUST negative—PCR BTM positive. The PCR DUST positive—PCR BTM negative responses were associated with lower bacterial loads in the test positive samples, compared to situations where *C*. *burnetii* DNA was detected in both the BTM and dust samples. This is consistent with Model 2 and reflects a low exposure of cows to bacteria from the environment and, possibly, an absence of shedding of bacteria by cows in the environment.

Thanks to the longitudinal study design with a constant time interval between sample collections, we were able to take the temporal correlation in farm statuses between sampling times into account. We propose a new model parameterization that allows evaluating disease dynamics through the estimation of an infection rate (τ_0_) and of a clearance rate (1-τ_1_) within a SIS (Susceptible-Infectious-Susceptible). Although the 2 prior distributions considered resulted in an estimated 10% difference in latent status prevalences, the estimated values for the probabilities of new infection (0.15 to 0.18) and infection persistence (0.91 to 0.93) were relatively close. This parameterization presents the further advantage of allowing the incorporation of previous herd data for the estimation of the latent (true) herd status. This can be helpful for diseases that are not eliminated easily and for which diagnostic tests have a poor sensitivity. This was the case for Q fever, but would also apply to other diseases of importance in farm animals such as paratuberculosis. To address this problem, other authors have developed Bayesian models with change points representing the transition from non-infected to infected [14, 16]. Our approach is simpler, but requires that the tests are carried out at equally spaced time intervals.

The different priors tested in the 2 models allowed us to reconsider the definition of the latent state. Our initial assumption was that it was very unlikely for a PCR to yield a positive result when there were no *C*. *burnetii* in the sample tested. Furthermore, because the bacteria are considered to be highly infectious, we had assumed that their presence was likely associated with the spread of the infection in the farms in which they were identified. However, relaxing the constraints on the prior for the specificity of PCR DUST was associated with a better model fit and a posterior mean specificity of 0.72 for PCR DUST. This therefore indicates that a PCR performed on DUST can be positive even though the herd is negative for the latent status. Our interpretation is that bacterial DNA was amplified from the sample, but, given the constraints imposed by the model and the data, these bacteria were not associated with a positive latent status. This latent status was determined from both the results of the 2 tests and the previous latent status. Regarding the latter, the transition from the previous to the current status was constrained by 2 model parameters representing new infections and cures. Both the probabilities of acquiring (0.15) and of curing (1–0.91 = 0.09) the infection were relatively small and resulted in between 5 and 6% ((1-π) τ_0_ = π (1-τ_1_)) of all herds acquiring and curing the infection between consecutive sampling times. This could have led to some herds positive for PCR DUST being classified as negative for the latent status, especially if they were negative for PCR BTM on the current (perfect specificity of PCR BTM) and next (small probability of cure) sampling times. Therefore, from an initial hypothesis where the identification of bacterial DNA was considered as significant from an epidemiological point of view, a model incorporating disease dynamics suggests that, in some cases, the presence of bacteria may not be sufficient to consider that *C*. *burnetii* are circulating between animals. While latent class analysis is usually performed to evaluate test characteristics and disease prevalence, it can also be used to investigate disease dynamics. In our case, it led to questioning the meaning of the latent status.

The two PCR tests appear to be complementary to identify contaminated farms, i.e. where *C*. *burnetii* is present. From Model 2, 92% of them were likely to be detected, in case the responses from both PCR tests were considered using a parallel reading. This increase in sensitivity associated with combining PCR tests responses (0.92 instead of 0.83 when considering the PCR BTM response alone) was related to their ability to detect very low bacterial levels in at least one sample (dust or milk). Thus, a perspective of this study could be to investigate the *C*. *burnetii* status of professionals (farmers, abattoir workers, veterinarians…) at risk of being exposed to the bacteria, and to assess the extent to which different levels of exposure within contaminated farms are associated with infection in humans. In this case using PCR DUST and PCR BTM would allow maximizing sensitivity.

The presence of *C*. *burnetii* on farm may not always be associated with active and persistent infections which contradicts the classical assumption of extreme infectiousness of the bacteria. When the objective is to detect the presence of shedder cows on farm, a PCR test performed on BTM should be preferred. When the objective is to detect the presence of bacteria on farm, whatever their epidemiological role, a PCR test based on the detection of *C*. *burnetii* DNA in dust samples collected with environmental swabs has shown good performance and could therefore be proposed, alone or in combination with PCR applied to BTM.

## Supporting Information

S1 Dataset Raw data used in this manuscript, including binary and continuous outcomes to both PCR applied to BTM and dust samples. (CSV)

S1 R Code R codes used in the analyses. (R)
